# Supplementary material for: Political endorsement by Nature and trust in scientific expertise during COVID-19
Source: Nat Hum Behav. 2023 Mar 20;7(5):696–706. doi: 10.1038/s41562-023-01537-5 (PMC10202798; doi:10.1038/s41562-023-01537-5)
Supplement: Supplementary file 1 — Supplementary Methods 1–3, Tables 1–4, Notes 1 and 2, and a complete copy of the survey instrument. [file 41562_2023_1537_MOESM1_ESM.pdf]

nature human behaviour

Article

https://doi.org/10.1038/s41562-023-01537-5

Political endorsement by *Nature* and trust in scientific expertise during COVID-19

In the format provided by the authors and unedited

Contents

1 Supplementary Methods **2**
1.1 Multiple Testing 2
1.2 Regression adjustment estimation with lasso 2
1.3 Experimental Results with the First 4,000 Responses 2

2 Supplementary Tables **3**

3 Supplementary Notes **7**
3.1 Subject Attention 7
3.2 Manipulation Check 7

References **8**

Survey Instrument **9**

1 Supplementary Methods

1.1 Multiple Testing

Table 2 reports results of adjusting analyses in the main text for multiple testing using sharpened False Discovery Rate (sharpened FDR) q-values[1][2].

As specified in the pre-analysis plan, I divide all ten outcomes into five mutually exclusive and collectively exhaustive sets, namely "stated trust in *Nature*", "endorsement persuasiveness", "demand for information", "climate Change", and "trust in scientists in general". *Within each set of outcomes*, I adjust for multiple testing for each of the two following families of hypotheses: "the treatment effect for Trump supporters is zero" and "the treatment effect for Biden supporters is zero". This groups 20 hypotheses about treatment effects into 10 clusters, of which two clusters, namely Trump supporters' and Biden supporters' demand for information (from *Nature*), are singletons containing only one hypothesis each and are thus *not* adjusted. As Table 2 makes clear, the corrections do not alter change the results in any meaningful way.

1.2 Regression adjustment estimation with lasso

During the experiment, a rich set of covariates were collected, including information on the subject's political ideology, age, gender, U.S. state of residence, race/ethnicity, education attainment, subject of post-secondary study (STEM or not), religion, residential setting (urban/suburb/rural), interest in current events, and interest in popular science reading. For simplicity and transparency, analyses presented in the main text do not make use of these information. In this appendix, I present treatment effect estimates with regression adjustments using these auxilary features to reduce estimator variance.

To efficiently select relevant covariates and regularize parameters for each outcome, I use lasso regression[3] to regularize the parameters, in accordance to my pre-analyzed plan. Specifically, I employ the lasso regression adjustment method proposed by Wager *et al*[4] to separately estimate the treatment effects on Biden supporters and on Trump supporters. The method combines unbiased estimation of treatment effects and regularized control of auxiliary features. The results are presented in Table 3. The adjustments reduce standard errors modestly and do not meaningfully alter the point estimates.

1.3 Experimental Results with the First 4,000 Responses

The pre-registration specified that the study would collect 4,000 responses and that the the experiment would end on 9 August 2021. During the experiment, Lucid Thoerem kept collecting data on 10 August 2021, which resulted in a larger-than-expected sample. The analyses presented in the main text are based on this larger-than-expected sample. In this Appendix, I analyze the first 4,000 responses collected, the last of which was collected on 9 August 2021. In adherence to the pre-registration, I exclude subjects who state that

they support neither Biden nor Trump from the 4,000 observations, yielding an analysis sample size of 3,657. Table 4 presents the resulting estimates. As expected, the estimates are almost identical to those from the larger sample presented in the main text.

2 Supplementary Tables

Table 1 Sample Breakdown by (Hypothetical) Presidential Vote Intention

	Number	Percentage
Definitely Biden	1,943	45.61%
Probably Biden	406	9.53%
Probably Trump	315	7.39%
Definitely Trump	1,221	28.66%
Someone else	375	8.80%
Total	4,260	100%

Note: only subjects who passed the attention check are included. he question asks who the subject would vote for if he or she "was to choose again". Question placed prior to treatment assignment.

Table 2 Multiple Testing Adjustments

Outcome z-score	$\widehat{CATE}$ Trump supporters [p-value] {SFDR q-value}	$\widehat{CATE}$ Biden supporters [p-value] {SFDR q-value}
Nature is informed when providing advice	-0.854 [0.000] {0.001}	0.108 [0.000] {0.001}
Nature is unbiased on contentious issues	-0.633 [0.000] {0.001}	0.0450 [0.151] {0.082}
Biden would have handled COVID better than Trump had he been president in 2020	0.0556 [0.085] {0.343}	0.0448 [0.061] {0.224}
Trump would have handled COVID better than Biden if he was still president now(2021)	-0.0241 [0.465] {0.441}	-0.0168 [0.485] {0.478}
Biden is better at making use of scientific knowledge for decision-making than Trump	0.0449 [0.204] {0.343}	0.0135 [0.478] {0.478}
Subject requests *Nature*'s article for info about vaccine efficacy against new variants	-0.285 [0.000] N/A^{+}	-0.048 [0.238] N/A^{+}
U.S. scientists are informed when providing advice	-0.130 [0.015] {0.008}	0.0485 [0.148] {0.421}
U.S. scientists are unbiased on contentious issues	-0.161 [0.002] {0.005}	0.0161 [0.604] {0.433}
"≥90%" of climate scientists agree human-caused climate change is real	-0.0461 [0.409] {1.000}	0.0265 [0.449] {1.000}
Human-caused climate change is real	-0.0232 [0.690] {1.000}	0.0147 [0.582] {1.000}

$N = 3,885$

$^{+}$ Multiple testing adjustment not applicable to hypotheses in "singleton" families.

p-values in the square brackets; Sharpened False Discovery Rate q-values in curly brackets.

All null hypotheses are testing are two-sided t tests.

Table 3 Regression Adjustment Estimates with Lasso Regression

Outcome z-score	$\widehat{CATE}$ Trump supporters (Robust s.e.)	$\widehat{CATE}$ Biden supporters (Robust s.e.)
Nature is informed when providing advice	-0.837<br>(0.0498)<br>p=0.000	0.120<br>(0.0292)<br>p=0.000
Nature is unbiased on contentious issues	-0.605<br>(0.0462)<br>p=0.000	0.0606<br>(0.0283)<br>p=0.030
Biden would have handled COVID better than Trump had he been president in 2020	0.0607<br>(0.0318)<br>p=0.061	0.0428<br>(0.0231)<br>p=0.072
Trump would have handled COVID better than Biden if he was still president now(2021)	-0.0335<br>(0.0318)<br>p=0.320	-0.0118<br>(0.0228)<br>p=0.631
Biden is better at making use of scientific knowledge for decision-making than Trump	0.0542<br>(0.0346)<br>p=0.112	0.0131<br>(0.0137)<br>p=0.326
Subject requests *Nature*'s article for info about vaccine efficacy against new variants	-0.271<br>(0.0444)<br>p=0.000	0.0534<br>(0.0395)<br>p=0.172
U.S. scientists are informed when providing advice	-0.119<br>(0.0525)<br>p=0.023	0.0573<br>(0.0313)<br>p=0.075
U.S. scientists are unbiased on contentious issues	-0.137<br>(0.0494)<br>p=0.007	0.0279<br>(0.0288)<br>p=0.324
"≥90%" of climate scientists agree human-caused climate change is real	-0.0483<br>(0.0559)<br>p=0.386	0.0294<br>(0.0342)<br>p=0.391
Human-caused climate change is real	-0.0042<br>(0.0545)<br>p=0.915	0.0153<br>(0.0262)<br>p=0.604
N	1,530	2,336

"$\widehat{CATE}$ Trump (Biden) supporters" is the estimated treatment effect for Trump (Biden) supporters. All treatment effect and standard errors are estimated using the lasso adjustment methods proposed by [4]. Sample includes 1,173 control Biden supporters, 1,176 treatment Biden supporters, 766 control Trump supporters, and 770 treatment Trump supporters. Outcomes are z-scores with mean 0 and standard deviation 1. All null hypotheses are testing are two-sided t tests.

Table 4 Experimental Results with the First 4,000 Responses

	$\widehat{CATE}$		
Outcome z-score	Trump supporters (Robust s.e.)	Biden supporters (Robust s.e.)	Baseline difference (Robust s.e.)
Nature is informed when providing advice	-0.852<br>(0.0534)<br>p=0.000	0.106<br>(0.0315)<br>p=0.001	-0.400<br>(0.0395)<br>p=0.000
Nature is unbiased on contentious issues	-0.612<br>(0.0514)<br>p=0.000	0.0430<br>(0.0322)<br>p=0.181	-0.670<br>(0.0410)<br>p=0.000
Biden would have handled COVID better than Trump had he been president in 2020	0.0546<br>(0.0329)<br>p=0.096	0.0459<br>(0.0244)<br>p=0.060	-1.647<br>(0.0289)<br>p=0.000
Trump would have handled COVID better than Biden if he was still president now(2021)	-0.0241<br>(0.0339)<br>p=0.478	-0.0123<br>(0.0244)<br>p=0.615	1.636<br>(0.0294)<br>p=0.000
Biden is better at making use of scientific knowledge for decision-making than Trump	0.0496<br>(0.0363)<br>p=0.172	0.0183<br>(0.0143)<br>p=0.200	-1.778<br>(0.0273)<br>p=0.000
Subject requests *Nature*'s article for info about vaccine efficacy against new variants	-0.267<br>(0.0477)<br>p=0.000	0.027<br>(0.0422)<br>p=0.527	-0.409<br>(0.0468)<br>p=0.000
U.S. scientists are informed when providing advice	-0.130<br>(0.0545)<br>p=0.017	0.0413<br>(0.045)<br>p=0.232	-0.768<br>(0.0452)<br>p=0.000
U.S. scientists are unbiased on contentious issues	-0.150<br>(0.0530)<br>p=0.005	0.0185<br>(0.0319)<br>p=0.562	-0.951<br>(0.0433)<br>p=0.000
"≥90%" of climate scientists agree human-caused climate change is real	-0.0306<br>(0.0574)<br>p=0.594	0.0282<br>(0.0359)<br>p=0.412	-0.612<br>(0.0478)<br>p=0.000
Human-caused climate change is real	-0.0394<br>(0.0596)<br>p=0.509	0.0224<br>(0.0273)<br>p=0.391	-0.984<br>(0.0466)<br>p=0.000

$N = 3,657$ $\widehat{CATE}$ Trump (Biden) supporters" is the estimated treatment effect for Trump (Biden) supporters. "Baseline difference" is the mean difference between Trump supporters and Biden supporters within the control group. Sample includes 1,102 control Biden supporters, 1,106 treatment Biden supporters, 722 control Trump supporters, and 727 treatment Trump supporters. Outcomes are z-scores with mean 0 and standard deviation 1. All estimates are from OLS estimation of a linear regression model, described in the Method section. Robust standard errors in parentheses. All null hypotheses are testing are two-sided t tests.

3 Supplementary Notes

3.1 Subject Attention

The analysis sample is the result of screening out inattentive respondents. A grid of questions are placed before the treatment and consists of three substantive questions and three attention checks. To pass the check, a participant must answer all three attention checks ”correctly”. The attention check asks if subjects agree or disagree with the following statements (1) ”Vermont is LESS populous than California. ” (passable answers: ”Strongly Agree”, ”Agree”, ”Neither Agree Nor Disagree”, or ”Don’t Know”) ; (2) ”Please select ‘Disagree”’ (passable answer: ”Disagree”); and (3)”Two plus one equals three”, (passable answers: ‘Strongly Agree” or ”Agree”). To make the attention screening less apparent to inattentive subjects, the survey mixes these questions with three substantive questions about subjects’ interests in science and current events. The check had a passing rate of 72.29% - of the 6,170 respondents who entered the survey, 1,710 failed the attention checks and were screened out from the study. Of the 4,460 subjects who passed the attention heck, 200 did not complete the survey and were excluded from the analyses.

3.2 Manipulation Check

The following manipulation check is placed after the last outcome question, near the end of the survey, to examine if the treatment is successful at informing subjects of *Nature*’s endorsement.

> Did the scientific journal Nature make any explicit political statements in support of any candidates in the run-up to the 2020 presidential election?

- No, they did not.
- Yes, they endorsed Joe Biden.
- Yes, they endorsed Donald Trump.
- Don’t know.

Experimental data shows that the treatment produced a large and statistically highly significant shift in the rate at which subjects answer ”Yes, they endorsed Joe Biden.”. Among Biden supporters, 68.79% treated subjects correctly answered that *Nature* endorsed Biden - a 43.47-percentage point shift ($p < 0.001$) relative to control Biden supporters, who answered correctly 25.32% of the time. The magnitude of the shift is remarkably similar among Trump supporters, albeit from a lower baseline. Treated Trump supporters answered correctly 58.57% of the time, 45 percentage points ($p < 0.001$) more frequent than the 13.58% baseline among control Trump supporters. Despite starting from different baselines, Biden supporters and Trump supporters were similarly receptive to the message - there is no statistically significant difference in the magnitude of the shift between Biden and Trump supporters ($p = 0.594$). Thus, any heterogeneity in treatment effects on outcomes is not due to differential compliance to the treatment.

References

[1] Anderson, M.L.: Multiple inference and gender differences in the effects of early intervention: A reevaluation of the abecedarian, perry preschool, and early training projects. Journal of the American Statistical Association **103**(484), 1481–1495 (2008)

[2] Benjamini, Y., Krieger, A.M., Yekutieli, D.: Adaptive linear step-up procedures that control the false discovery rate. Biometrika **93**(3), 491–507 (2006)

[3] Tibshirani, R.: Regression shrinkage and selection via the lasso. Journal of the Royal Statistical Society: Series B (Methodological) **58**(1), 267–288 (1996)

[4] Wager, S., Du, W., Taylor, J., Tibshirani, R.J.: High-dimensional regression adjustments in randomized experiments. Proceedings of the National Academy of Sciences **113**(45), 12673–12678 (2016)

Consent

RESEARCH INFORMATION

DESCRIPTION: You are invited to participate in a research study on public attitudes . You will be asked to answer some questions on, and read some short paragraphs about social affairs.

TIME INVOLVEMENT: Your participation will take approximately 9 minutes.

RISKS AND BENEFITS: There is no risks associated with this study. We cannot and do not guarantee or promise that you will receive any benefits from this study.

PAYMENT: You will be compensated based on the amount agreed upon between you and the panel company.

PARTICIPANT'S RIGHTS: If you have read this form and have decided to participate in this project, please understand your participation is voluntary and you have the right to withdraw your consent or discontinue participation at any time without penalty or loss of benefits to which you are otherwise entitled. The alternative is not to participate. You have the right to refuse to answer particular questions. The results of this research study may be presented at scientific or professional meetings or published in scientific journals. Your individual privacy will be maintained in all published and written data resulting from the study.

CONTACT INFORMATION:
Questions: If you have any questions, concerns or complaints about this research, its procedures, risks and benefits, contact the Protocol Director, Floyd Zhang at [redacted].
Independent Contact: If you are not satisfied with how this study is being conducted, or if you have any concerns, complaints, or general questions about the research or your rights as a participant, please contact the Stanford Institutional Review Board (IRB) to speak to someone independent of the research team at 650-723-2480 or email at IRB2-Manager@lists.stanford.edu or toll free at 1-866-680-2906. You can also write to the Stanford IRB, Stanford University, 1705 El Camino Real, Palo Alto, CA 94306.

Due to the coronavirus public health emergency, the federal government has issued a Declaration that may limit your right to sue if you are injured or harmed while participating in this COVID-19 study. If the Declaration applies, it limits your right to sue researchers, healthcare providers, any study sponsor, manufacturer, distributor or any other official involved with the study. However, the federal government has a program that may provide compensation to you or your family if you experience serious physical injuries or death. To find out more about this “Countermeasures Injury Compensation Program” please go to https://www.hrsa.gov/cicp/about/index.html or call 1-855-266-2427.

The extra copy of this consent form is for you to keep.

*If you agree to participate in this research, please **click the box below** and complete the questionnaire.*

- I have read the information above and consent to participate.
- I do not consent to participate.

Pre-Treatmet Political Questions

First, we would like to ask some questions about yourself.

Generally speaking, do you usually think of yourself as a Democrat, a Republican, an independent, or what?

- Democrat
- Republican
- Independent
- Other party

Would you call yourself a strong Democrat or a not very strong Democrat?

- Strong
- Not very strong

Would you call yourself a strong Republican or a not very strong Republican?

- Strong
- Not very strong

Do you think of yourself as closer to the Republican Party or the Democratic Party?

- Closer to Republican
- Closer to Democratic

Who did you vote for in the past presidential election in 2020?

- Donal Trump
- Joe Biden
- Someone else
- Did not vote

If you were to choose again, and the major-party candidates were still Joe Biden the Democrat and Donald Trump the Republican, for whom would you vote?

- Definitely Trump
- Probably Trump
- Probably Biden
- Definitely Biden
- Someone else

Where would you place yourself on this liberal-conservarive scale?

- Extremely liberal
- Liberal
- Slightly liberal
- Moderate
- Slightly conservative
- Conservative
- Extremely conservative

Prior

Nature is one of the most-cited scientific journals in the world. How likely do you think it is that *Nature* officially endorsed one of the candidates in the 2020 presidential election?

- They definitely did
- Very likely
- Somewhat likely
- Not very likely
- Not likely at all

Hypothetically, suppose *Nature* made an endorsement. Who would they endorse?

- Definitely Biden
- Probably Biden
- Equally likely to endorse Biden or Trump
- Probably Trump
- Definitely Trump

Attention Check 1

Do you agree or disagree with the following statements?

	Strongly Agree	Agree	Neither Agree Nor Disagree	Disagree	Strongly Disagree	Don't Know
I follow news about current events closely	○	○	○	○	○	○
Vermont is LESS populous than California	○	○	○	○	○	○
I am worried about the vaccines' effectiveness against emerging COVID-19 variants	○	○	○	○	○	○
Please select "Disagree"	○	○	○	○	○	○

Two plus one equals three	○	○	○	○	○	○
I enjoy popular science readings	○	○	○	○	○	○

Attention Check 2

People are very busy these days and many do not have time to follow what goes on in the government. **We are testing whether people read questions**. To show that you've read this much, answer **both** "extremely interested" **and** "very interested."

- ☐ Extremely interested
- ☐ Very interested
- ☐ Moderately interested
- ☐ Slightly interested
- ☐ Not interested at all

Treatment/Control

Please read the following short text
before answering the next set of questions

Scientific journal *Nature* endorsed Joe Biden

- Weeks before the 2020 presidential election, ***Nature***'s editorial board officially endorsed Joe Biden, citing:
    - Donald Trump's pandemic response had been "disastrous";
    - Biden, unlike Trump, would listen to science, and thus;

- Biden would handle the COVID-19 pandemic better.

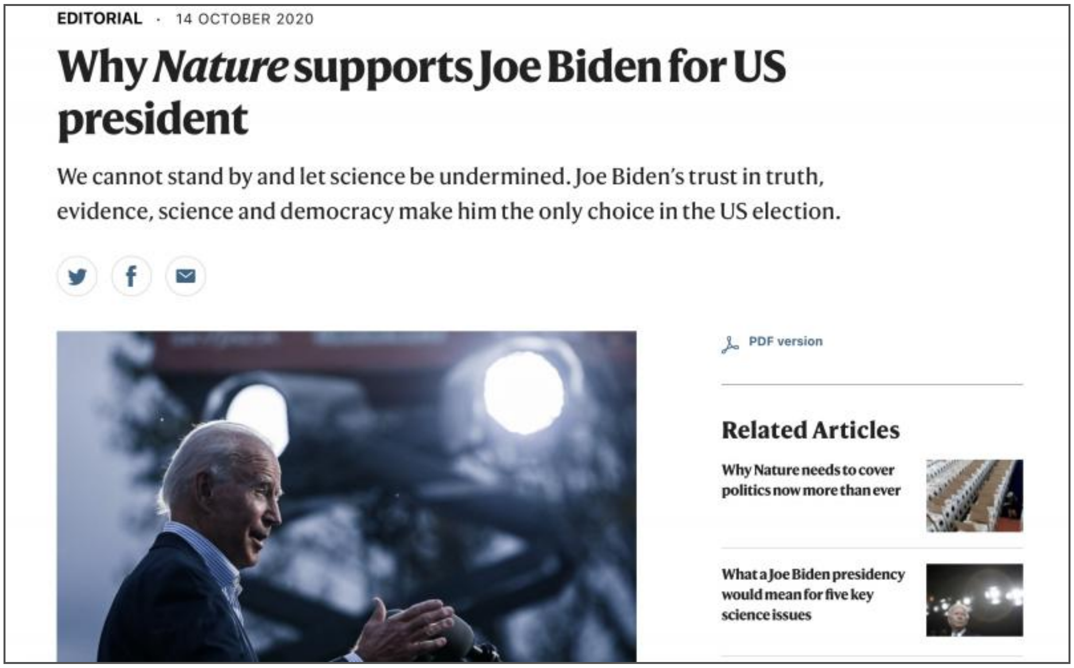

EDITORIAL · 14 OCTOBER 2020

Why *Nature* supports Joe Biden for US president

We cannot stand by and let science be undermined. Joe Biden's trust in truth, evidence, science and democracy make him the only choice in the US election.

PDF version

Related Articles

Why Nature needs to cover politics now more than ever

What a Joe Biden presidency would mean for five key science issues

(Reference: The full endorsement can be found here: https://www.nature.com/articles/d41586-020-02852-x)

- ***Nature*** is one of the most-cited and most prestigious peer-reviewed scientific journals in the world.

nature

Please read the following short text before answering the next set of questions

Scientific journal *Nature* unveiled its new design

- In October 2019, ***Nature***'s editorial board unveiled a new design for the scientific journal, including:
  - New print and digital designs:
  - A new logo.
- The editors hope the redesign will help the journal to communicate research with style and greater clarity.

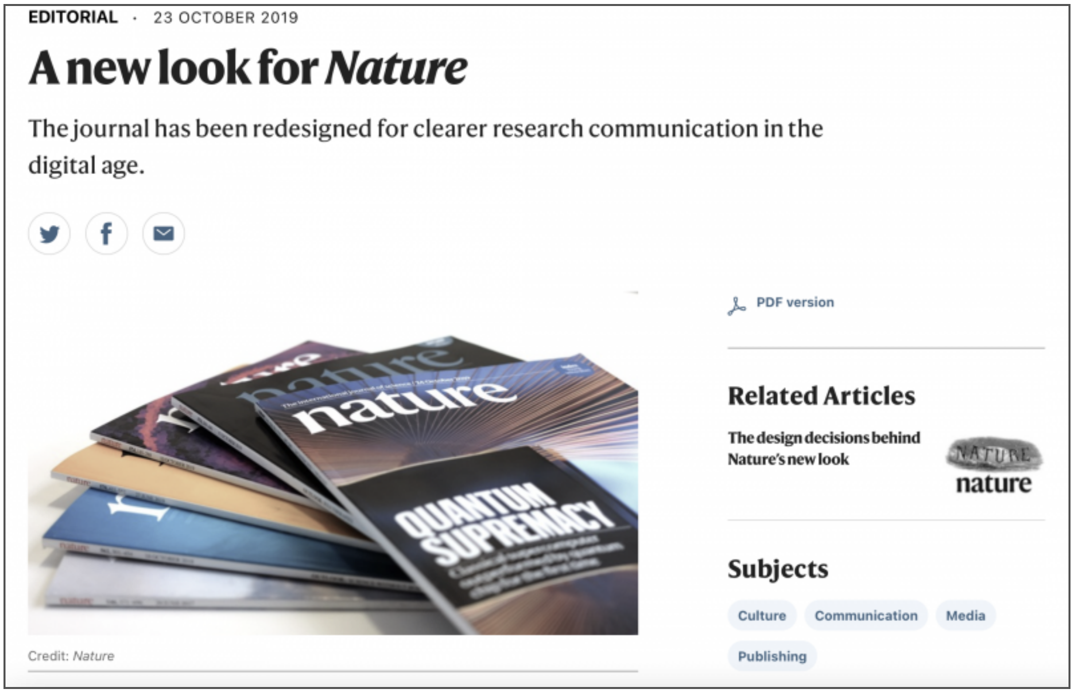

EDITORIAL · 23 OCTOBER 2019

A new look for *Nature*

The journal has been redesigned for clearer research communication in the digital age.

PDF version

Related Articles

The design decisions behind Nature's new look

Subjects

Culture Communication Media Publishing

Credit: *Nature*

(Reference :The full announcement can be found here: https://www.nature.com/articles/d41586-019-03167-2)

- ***Nature*** is one of the most-cited and most prestigious peer-reviewed scientific journals in the world.

nature

In your opinion, how **informed** are editors of the journal *Nature*, when it comes to providing advice on science-related issues facing the society?

- Extremely informed
- Fairly informed
- Somewhat informed
- Not very informed
- Not informed at all

When contentious or divisive issues are concerned, how much confidence do you have in the editorial board of *Nature* to provide their **unbiased** opinions to

the public, to the best of their knowledge?

- A great deal
- A lot
- A moderate amount
- A little
- None at all

Policies and Politics

Next, we would like to ask a few questions on public policies and politics

Recall early 2020, when the COVID-19 outbreak began and Donald Trump was president. Had Joe Biden been president back then, would he have done a better job handling the pandemic than Trump did?

- Biden would have done much worse
- Biden would have done worse
- Biden would have done somewhat worse
- Biden would have done exactly the same
- Biden would have done somewhat better
- Biden would have done better
- Biden would have done much better

Now think instead about the present. If Donald Trump was president now, would he have done a better job handling COVID than Joe Biden has done since inauguration?

- Trump would have done much worse
- Trump would have done worse
- Trump would have done somewhat worse
- Trump would have done exactly the same
- Trump would have done somewhat better
- Trump would have done better
- Trump would have done much better

Which candidate of the 2020 presidential election, Joe Biden or Donald Trump, is better at making appropriate use of scientific knowledge for decision-making in general?

- Definitely Biden
- Probably Biden
- Maybe Biden
- Exactly the same
- Maybe Trump
- Probably Trump
- Definitely Trump

Demand for information

Stay Informed About Vaccine Efficacy Against New COVID Variants

- Emerging COVID-19 variants have been discovered around the globe. Many of the new variants are fast-spreading and raise public concerns about possible diminished vaccine effectiveness.

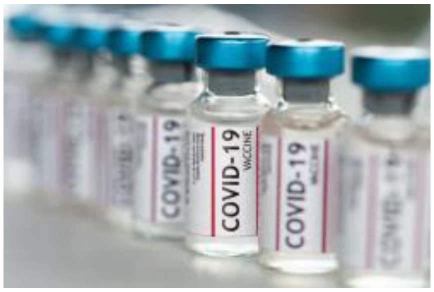

Credit: MarsBars/Getty

- To help you stay informed, we would like to share with you some **links easy-to-read articles on** what scientists know about these **new variants and how well available vaccines perform against them.**

(Note: reading the articles is NOT required for receiving your payment. You may select your preferred sources and save the links to read them later.)

Please select ALL sources you would like to read from. You may select multiple sources.

- ☐ I would like to read an article from *Nature*
- ☐ I would like to read an article from the Mayo Clinic
- ☐ I would like to read an article from news media websites
- ☐ I do not want to read about the new variants or vaccine effectiveness

Are you sure you do not want to learn about the new variants and how effective available vaccines are against them?

Again, reading the articles is NOT required for receiving your payment. You may bookmark or save the links to read them later.

Please check ALL sources you would like to read from. You may select multiple sources.

- ☐ I would like to read an article from *Nature*

- ☐ I would like to read an article from the Mayo Clinic
- ☐ I would like to read an article from news media websites
- ☐ I am sure that I do not want to read about the new variants or vaccine effectiveness

For your information, we recommend the *Nature* article below.

What scientists know about new, fast-spreading coronavirus variants - *Nature*

Again, reading the article is NOT required for receiving your payment. You can bookmark or save the page for later.

[Note: More information on COVID-19 for the American public available at: https://www.cdc.gov/coronavirus/2019-ncov/index.html]

For your information, we recommend the Mayo Clinic articles below.

What is a COVID-19 variant strain? - Mayo Clinic

COVID-19 Variants: what's the concern? - Mayo Clinic

Again, reading the article is NOT required for receiving your payment. You can bookmark or save the page for later.

[Note: More information on COVID-19 for the American public available at: https://www.cdc.gov/coronavirus/2019-ncov/index.html]

For your information, we recommend the Yahoo News article below.

Delta virus variant, which took over in the UK and threatens the US, doubles the

risk of hospitalization, new data says. But vaccines are still effective. - Yahoo News

Again, reading the article is NOT required for receiving your payment. You can bookmark or save the page for later.

[Note: More information on COVID-19 for the American public available at: https://www.cdc.gov/coronavirus/2019-ncov/index.html]

For your information, we recommend the articles below.

What scientists know about new, fast-spreading coronavirus variants - *Nature*

What is a COVID-19 variant strain? - Mayo Clinic

COVID-19 Variants: what's the concern? - Mayo Clinic

Again, reading the article is NOT required for receiving your payment. You can bookmark or save the page for later.

[Note: More information on COVID-19 for the American public available at: https://www.cdc.gov/coronavirus/2019-ncov/index.html]

For your information, we recommend the articles below.

What scientists know about new, fast-spreading coronavirus variants - *Nature*

Delta virus variant, which took over in the UK and threatens the US, doubles the risk of hospitalization, new data says. But vaccines are still effective. - Yahoo News

Again, reading the article is NOT required for receiving your payment. You can bookmark or save the page for later.

[Note: More information on COVID-19 for the American public available at: https://www.cdc.gov/coronavirus/2019-ncov/index.html]

For your information, we recommend the articles below.

What is a COVID-19 variant strain? - Mayo Clinic

COVID-19 Variants: what's the concern? - Mayo Clinic

Delta virus variant, which took over in the UK and threatens the US, doubles the risk of hospitalization, new data says. But vaccines are still effective. - Yahoo News

Again, reading the article is NOT required for receiving your payment. You can bookmark or save the page for later.

[Note: More information on COVID-19 for the American public available at: https://www.cdc.gov/coronavirus/2019-ncov/index.html]

For your information, we recommend the articles below.

What scientists know about new, fast-spreading coronavirus variants - *Nature*

What is a COVID-19 variant strain? - Mayo Clinic

COVID-19 Variants: what's the concern? - Mayo Clinic

Delta virus variant, which took over in the UK and threatens the US, doubles the risk of hospitalization, new data says. But vaccines are still effective. - Yahoo News

Again, reading the article is NOT required for receiving your payment. You can bookmark or save the page for later.

[Note: More information on COVID-19 for the American public available at: https://www.cdc.gov/coronavirus/2019-ncov/index.html]

Climate Change

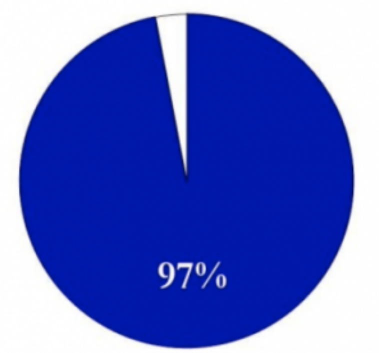

According to the editorial board of ***Nature***, **97% of climate scientists agree that climate change is both real and caused by human activities.**

nature

What share of climate scientists **do YOU think** agree that climate change is both real and caused by human activities?

- ○ <10%
- ○ 10-29%

- 30-49%
- 50-69%
- 70-89%
- ≥90%

Do you think human activity is causing changes to the world's climate?

- Definitely
- Probably
- Maybe
- Probably not
- Definitely not

Trust in Science

In general, how **informed** are **U.S. scientists** when it comes to providing advice on science-related issues facing the society?

- Extremely informed
- Fairly informed
- Somewhat informed
- Not very informed
- Not informed at all

When contentious or divisive issues are concerned, how much confidence do you have in **U.S. scientists** to provide their **unbiased** opinions to the public, to the best of their knowledge?

- A great deal
- A lot
- A moderate amount
- A little
- None at all

Manipulation Check

Did the scientific journal *Nature* make any explicit political statements in support of any candidates in the run up to the 2020 presidential election?

- No, they did not.
- Yes, they endorsed Joe Biden.
- Yes, they endorsed Donald Trump.
- Don't know

Do you remember what *Nature* said about Joe Biden? Check ALL you can recall. Feel free to answer "I don't know or remember any" if you can't recall any.

- He would push for more generous economic stimulus packages
- He would listen to science
- He would handle COVID better
- He would build a coalition of allies against China and Russia
- He would provide more funding for STEM education
- He would provide more funding for K-12 education
- He would push for a "Green New Deal"
- I don't know or remember any

Demographic

Finally, we have a few more questions about yourself.

How old are you?

- Under 18
- 18-24 years old
- 25-34 years old
- 35-44 years old
- 45-54 years old
- 55-64 years old
- 65+ years old

What's your gender?

- Male
- Female
- Others

In which state do you currently reside?

What is the highest level of education you have completed?

- Did not graduate high school

- High school graduate or GED
- Began college, no degree
- Associates or technical degree
- Bachelor’s degree
- Postgraduate or professional degree

What is your postgraduate or professional degree in? Check all that apply.

- Science
- Engineering
- Social Sciences
- Arts and Humanities
- Medicine
- Public Health
- Law
- Business
- Public Policy
- Other

What is your bachelor's degree in?

- Science
- Engineering
- Social Sciences
- Arts and Humanities
- Business
- Public Policy
- Other

What religious group do you consider yourself affiliated with?

- Mainline Protestant
- Historically black Protestant
- Evangelical Protestant
- Catholic
- Other Christian
- Jewish
- Muslim
- Other religion or faith
- Atheist
- Agnostic
- Nothing in particular

Which of the following best describes the area you live in?

- Urban – Densely populated, city or large town
- Suburban – Mainly residential, bordering a city or large town
- Rural – Sparsely populated, small town or village

Are you of Hispanic, Latino, or Spanish origin?

- No
- Yes

What is your race/ethnicity?

- Black or African American

- White
- American Indian or Alaska Native
- Asian
- Native Hawaiian or Other Pacific Islander
- Other

Powered by Qualtrics
