# Supplementary material for: Political endorsement by Nature and trust in scientific expertise during COVID-19
Source: Nat Hum Behav. 2023 Mar 20;7(5):696–706. doi: 10.1038/s41562-023-01537-5 (PMC10202798; doi:10.1038/s41562-023-01537-5)
Supplement: Supplementary file 2 — Reporting Summary [file 41562_2023_1537_MOESM2_ESM.pdf]

nature portfolio

Corresponding author(s): Floyd Jiuyun Zhang

Last updated by author(s): Jan 24, 2023

Reporting Summary

Nature Portfolio wishes to improve the reproducibility of the work that we publish. This form provides structure for consistency and transparency in reporting. For further information on Nature Portfolio policies, see our Editorial Policies and the Editorial Policy Checklist.

Statistics

For all statistical analyses, confirm that the following items are present in the figure legend, table legend, main text, or Methods section.

n/a	Confirmed	
☐	☒	The exact sample size (n) for each experimental group/condition, given as a discrete number and unit of measurement
☒	☐	A statement on whether measurements were taken from distinct samples or whether the same sample was measured repeatedly
☐	☒	The statistical test(s) used AND whether they are one- or two-sided<br>*Only common tests should be described solely by name; describe more complex techniques in the Methods section.*
☐	☒	A description of all covariates tested
☐	☒	A description of any assumptions or corrections, such as tests of normality and adjustment for multiple comparisons
☐	☒	A full description of the statistical parameters including central tendency (e.g. means) or other basic estimates (e.g. regression coefficient) AND variation (e.g. standard deviation) or associated estimates of uncertainty (e.g. confidence intervals)
☐	☒	For null hypothesis testing, the test statistic (e.g. F, t, r) with confidence intervals, effect sizes, degrees of freedom and P value noted<br>*Give P values as exact values whenever suitable.*
☒	☐	For Bayesian analysis, information on the choice of priors and Markov chain Monte Carlo settings
☒	☐	For hierarchical and complex designs, identification of the appropriate level for tests and full reporting of outcomes
☒	☐	Estimates of effect sizes (e.g. Cohen's d, Pearson's r), indicating how they were calculated

Our web collection on statistics for biologists contains articles on many of the points above.

Software and code

Policy information about availability of computer code

Data collection	I used Qualtrics XM to program and to distribute the experimental questionnaire, and to collect the resulting data.
Data analysis	I analyzed and visualized my data in Stata MP 14 and RStudio 1.4. In particular, the lasso regression adjustments are implemented using the crossEstimation package (https://github.com/swager/crossEstimation, the latest version as of Jan. 01 2023, last updated Feb. 20 2017) developed by Wager et al (2016).

For manuscripts utilizing custom algorithms or software that are central to the research but not yet described in published literature, software must be made available to editors and reviewers. We strongly encourage code deposition in a community repository (e.g. GitHub). See the Nature Portfolio guidelines for submitting code & software for further information.

Data

Policy information about availability of data

All manuscripts must include a data availability statement. This statement should provide the following information, where applicable:

- Accession codes, unique identifiers, or web links for publicly available datasets
- A description of any restrictions on data availability
- For clinical datasets or third party data, please ensure that the statement adheres to our policy

All data used in this study is generated specifically for the study during the experiment. The author will provide the dataset to editors and/or reviewers during the review process upon request. If accepted, the author will make the dataset publicly available on Harvard Dataverse before publication .

Human research participants

Policy information about studies involving human research participants and Sex and Gender in Research.

Reporting on sex and gender	I collected gender information by asking the following question in my survey questionnaire: "What's your gender?". The response options are "Female", "Male", and "Others". In my analysis sample (N=4,260), 2,257 (52.98%) identify as "Female"; 1,982 (46.53%) identify as "Male;"21 (0.49%) identify as "Others". The only place this information is used is in Appendix A, where I control for demographics. There's no gender-based analysis, since the heterogeneity of interest is prior political opinions given the research questions.
Population characteristics	I collected information about participants' gender, age, race, education, state of residence, political opinions, etc., in the survey questionnaire. The distribution is presented in Tables 1 and 2 of the manuscript. The sample is largely representative of the U.S. adult population, but Biden supporters seem to be over-represented relative to Trump supporters.
Recruitment	The respondents were recruited via Lucid Theorem, who aggregate respondents from various primary sources (online marketing panels. etc.) . Respondents were asked to share their opinions in "a survey about current events" in exchange for a monetary payment. The respondents signified consent by agreeing to a consent form and were screened for attention. These recruitment steps could introduce bias, but the resulting analysis sample is fairly represented of the U.S. adult population in terms of demographics (Table 1). The sample does not seem to be representative in terms of politics, with Biden supporters overrepresented. This does not introduce biased to the results since all estimates are conditional on prior political opinions. Given these, I think the estimates should be reasonably representative.
Ethics oversight	Stanford University IRB. (Protocol number: IRB-60462)

Note that full information on the approval of the study protocol must also be provided in the manuscript.

Field-specific reporting

Please select the one below that is the best fit for your research. If you are not sure, read the appropriate sections before making your selection.

☐ Life sciences ☒ Behavioural & social sciences ☐ Ecological, evolutionary & environmental sciences

For a reference copy of the document with all sections, see nature.com/documents/nr-reporting-summary-flat.pdf

Behavioural & social sciences study design

All studies must disclose on these points even when the disclosure is negative.

Study description	This is a quantitative experimental study with online participants. The experiment take the form of an online survey, with randomized information about Nature's endorsement of Joe Biden. Participants are then asked about their political and scientific attitudes. Statistical analyses are conducted to estimate the causal effect of seeing the endorsement on participants's views toward the journal Nature, U.S. scientists, Joe Biden, and Donald Trump.
Research sample	An online sample collected through Lucid Theorem that is broadly representative of the U.S. adult population in terms of age, sex, race, education level, and region of residence. The sample is chosen because of its demographic representativeness and ease of access.
Sampling strategy	Lucid Theorem constructs sample representative of the U.S. adult population from a pool of online respondents. The sample size is based on back-of-envelope calculation using data I collected in a pilot study.
Data collection	The respondents are given link to my Qualtrics questionnaire. Qualtrics records their responses. The experimental process is online and automated. No researcher is present when subjects go through the experiment.
Timing	July 28 - August 10, 2021
Data exclusions	375 completed responses (8.80% of the sample) are excluded because the subjects stated that they would vote for "someone else" (that is, neither Biden nor Trump). This is because their political positions don't have clear interpretation. I committed to this exclusion rule in my pre-registration /pre-analysis plan. See "Method" section in my manuscript for references to my pre-registration /pre-analysis plan.
Non-participation	221 subjects who did not choose to agree to the consent document were screened out automatically prior to taking the survey. 1,710 of the consenting subjects are removed because they failed the attention check (See "Subject attention" subsection of the "Method" section). Of the 4,460 who consented and passed the attention check, exactly 200 did not complete the questionnaire.
Randomization	Randomization is implemented via Qualtrics randomizer. Before randomization, the survey asked each participant who would they vote for if they were to choose again between Biden and Trump. There are five response options to the question "Definitely Biden",

"Probably Biden", "Definitely Trump", "Probably Trump", and someone else. The treatment is randomized within each of these five blocks to ensure finite sample balance. One half of the subjects in each block receive the treatment.

Reporting for specific materials, systems and methods

We require information from authors about some types of materials, experimental systems and methods used in many studies. Here, indicate whether each material, system or method listed is relevant to your study. If you are not sure if a list item applies to your research, read the appropriate section before selecting a response.

Materials & experimental systems

n/a	Involved in the study	
☒	☐	Antibodies
☒	☐	Eukaryotic cell lines
☒	☐	Palaeontology and archaeology
☒	☐	Animals and other organisms
☒	☐	Clinical data
☒	☐	Dual use research of concern

Methods

n/a	Involved in the study	
☒	☐	ChIP-seq
☒	☐	Flow cytometry
☒	☐	MRI-based neuroimaging
